# Special Issue “Fundamental and Practical Perspectives in Regenerative Medicine: Proceedings of the VI National Congress of Regenerative Medicine (2024)”

**DOI:** 10.3390/ijms27125610

**Published:** 2026-06-22

**Authors:** Pavel I. Makarevich, Vsevolod A. Tkachuk

**Affiliations:** 1 Center for Regenerative Medicine, Medical Research and Education Institute, Lomonosov Moscow State University, 27-10, Lomonosovskiy Avenue, Moscow 119192, Russia; tkachuk@fbm.msu.ru; 2 Faculty of Medicine, Medical Research and Education Institute, Lomonosov Moscow State University, 27-1, Lomonosovskiy Avenue, Moscow 119192, Russia

## 1. Field Outreach and General Overview of Methodology

The recent rapid advances in technological pipelines are urging faster and riskier translational moves from research to practice in regenerative medicine. Accumulated field experience has allowed certain barriers and limitations that existed for decades to be lifted to facilitate rapid availability for the patient. However, our article collection illustrates that despite the increasing need for practical achievements, which will be numerous and impressive, the majority of researchers are focusing on understanding the basic principles behind regeneration and tissue renewal.

Another hallmark of the currently evolving approaches is the wider and deeper use of genetic and transcriptomic analyses to describe novel mechanisms and analyze the regulation of the cell processes involved in regeneration at unprecedented resolution. These methods shifted from genomics to transcriptomics during the last decade, and the wider availability of these methods increased the number of studies in this field. In the present collection, approximately 2/3 of the works involve the use of transcriptomic analysis or genetic manipulation via viral vectors to modify or investigate cellular functions.

The article collection we cover below provides a wide overview of the field, and we summarize the main future research directions that may be of interest and were highlighted by the contributors.

## 2. Highlights of Prospective Directions

### 2.1. Genetic Technologies Provide Clues, Predict Targets, and Make Way for Novel Therapies

Overall, the use of transcriptomic and genomic methods has been in the spotlight since their wider introduction in cell biology and animal model studies. Nevertheless, in addition to high-throughput descriptive data, these methods have become effective in predicting molecular targets that have been successfully validated in further gain- and loss-of-function models of regenerative processes. Interestingly, these novel pipelines are not limited to yielding differentially expressed genes but cover numerous physiological aspects—from aging and frailty to epimorphic regeneration—that may occur in the human body as well, e.g., in the endometrium [1] and distal phalanx [2]. The most important hallmark of this novel field is its rapid and open-access expansion, with the majority of groups depositing the obtained datasets and bioinformatical algorithms into repositories, allowing other researchers to re-analyze them. The described “open science” model involves collective effort for deeper investigations and the identification of targets via customized pipelines. Indeed, one may not have enough human resources or time to validate every target or pathway that can be obtained from a strong enough RNA sequencing set—so making the dataset pubic is a great contribution to the field. More and more top peers are using others’ datasets for groundbreaking discoveries in the field of regenerative biology.

Another tool for studying regeneration is manipulation using viral or non-viral vectors, which allows the validation of predicted targets and establishes a basis for novel therapies. In our article collection, only one by Sattarov et al. is devoted to classical gene replacement therapy using a adeno-associated viral system to treat hemophilia B. In other instances, viral and non-viral systems were used to develop tissue-engineered constructs with regenerative potential, functionalize the MSC secretome, or downregulate desired targets—NOTCH1 and 2.

This methodology, along with experiments that describe cell fate (lineage tracing) and transcriptomic profile (single-cell RNA sequencing), will yield novel data on the course of events in normal and regenerating organs in mammals and especially in humans. Once a significant amount of comparative data have been accumulated, we may expect the establishment of new targets underestimated using conventional cell biology and biochemistry methods.

**Figure 1:**
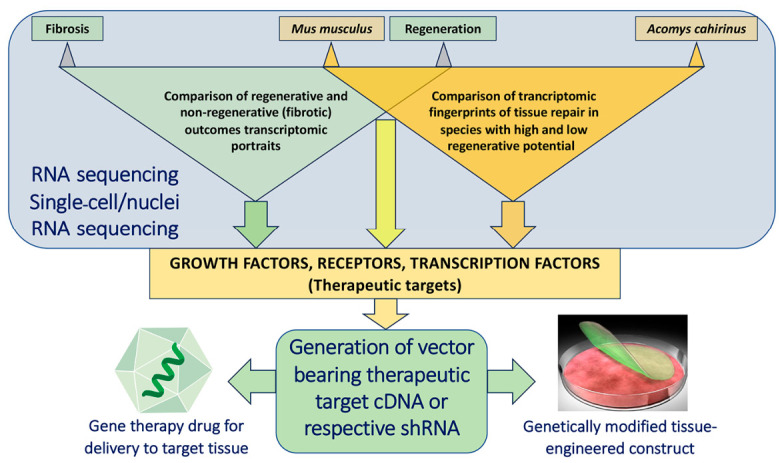
Schematic representation of comparative methodology using genetic technologies (e.g., RNA sequencing) and its application to establish therapeutic targets in regenerative medicine.

In our previous editorial, we focused on a comparative methodology to investigate the factors that underlie successful regeneration or fibrosis in the same tissue depending on damage severity [2]. This allows an understanding of the mechanistic differences between two mutually exclusive pathways (regeneration and fibrosis) and the examination of the spatiotemporal dynamics that precede these outcomes. Another way to advance is through the comparison of closely related species that regenerate vs. ones who fail; the latest examples are provided in numerous works on *Acomys* spp., which possess impressive regenerative capability in multiple organs—the skin, heart, liver, kidneys, and cartilage [3,4,5]. Once comparative analysis is performed of the *Acomys* vs. *Mus* settings, where the latter represents a species prone to fibrosis, we may determine the molecular pathways that stimulate regeneration (or block fibrosis) to use them as potential therapeutic targets (Figure 1).

Based on these results, one may expect the creation of a new field of gene therapy that allows the fine-tuning and regulation of regenerative processes—ranging from limiting scar formation (by suppressing fibrosis) to body part and limb re-growth, at least in an experimental setting.

### 2.2. Tissue Engineering Dominates and Utilizes Genetic Technologies

The last decade has seen a rapid rise in the use of tissue engineering and bioprinting in the purely experimental area of the biomedicine to translational framework. Despite obvious achievements in CAR T-cell therapy as a tool for cancer treatment, tissue engineering is taking the lead in regenerative medicine.

Progress in deconstructing the niche concept and—in particular—the understanding of stromal niche organization and modus operandi has resulted in a paradigm shift. We cannot expect a stem cell or MSC “dropped” to damaged tissue via injection or topical application to display its full regenerative program. Thus, tissue engineering is used to clarify the appropriate context prior to delivery or to rebuild “tissue blocks” that can be utilized for larger shapes. Tissue engineering has demonstrated diversity, ranging from 3D bioprinting, which shows great potential to achieve unprecedented positional accuracy, to self-assembling spheroids and cell sheets, which provide feasible and versatile platforms for a wide array of applications. It should be noted that genetically modified constructs are the point where gene delivery converges with tissue engineering, to provide constructs that are superior to conventional constructs. This approach largely enhances therapeutic efficacy and allows the functionalize of the construct’s paracrine activity for the appropriate application modality.

One should also acknowledge the potential of decellularization technologies applied to yield functional tissue-specific matrices that can be repopulated ex vivo. Indeed, taking into account the positional and functional roles of extracellular matrix proteins and the ligand–receptor interactions required for stem cell survival and differentiation, these cell-free scaffolds represent a feasible tool for both the modeling and generation of constructs with a natural matrix composition and structure. An article by Bogomolova et al. demonstrates that the cross-organ application of decellularized matrices has prospects for demanding cell types such as islet cells. Overall, once certain tissues are available (e.g., lung tissues), they can be used to derive a decellularized matrix that can be repopulated with other tissue’s cells and will function both in vitro and in vivo.

The application of genetic technologies in this area has provided important data that cannot be obtained via morphological or functional means. The RNA sequencing of tissue-engineered objects (e.g., spheroids or cell sheets) resulted in the establishment of the regulatory pathways responsible for stem/progenitor cell expansion [6], epigenetic stability and renewal [7], self-organization, and lineage commitment, which are hard to predict due to the significant heterogeneity among tissue-like systems. Generally, future insights gained in the field will e connected to genetic technologies and -omics analyses that provide comprehensive data and identify points for regeneration control, including potential safety concerns that may be predicted prior to pre-clinical or clinical translation.

## 3. Conclusions

The convergence of genetic technologies and comparative methodologies is a promising approach for yielding novel sets of unique data for bioinformatical processing and the establishment of relevant therapeutic targets. The latter may include pivotal molecules that switch tissue repair or regulate tissue re-growth after damage as well as cell (sub)populations or physical factors (e.g., stiffness, viscosity, tension) that govern events after damage.

From the variety of methods that can be used for translational studies, gene therapy is a method of choice as it allows targeted delivery to up- or downregulate desired factors in the tissue or the creation of a tissue-engineered construct used for further transplantation (Figure 1).

We believe that operating via the proposed pipeline is a prospective way to uncover physiologically justified targets and use them for the development of novel biomedical treatments. This biomimetic-based method relies on hints from naturally occurring healing and regeneration, and this comparative methodology is an effective route to identify the factors involved in the sequence of events leading to a desired or pathological outcome after damage. Hopefully, this method will facilitate and drive progress in regenerative medicine and physiology to accomplish the long-term goals of the effective re-growth of human tissue and the restoration of organ function.
